# Photographic Capture-Recapture Sampling for Assessing Populations of the Indian Gliding Lizard *Draco dussumieri*

**DOI:** 10.1371/journal.pone.0055935

**Published:** 2013-02-13

**Authors:** Rachakonda Sreekar, Chetana B. Purushotham, Katya Saini, Shyam N. Rao, Simon Pelletier, Saniya Chaplod

**Affiliations:** 1 Key Laboratory of Tropical Forest Ecology, Xishuangbanna Tropical Botanical Garden, Chinese Academy of Sciences, Mengla, Yunnan, China; 2 Agumbe Rainforest Research Station, Agumbe, Karnataka, India; 3 National Centre for Biological Sciences, Tata Institute of Fundamental Research, Bangalore, Karnataka, India; 4 Colorado State University, Fort Collins, Colorado, United States of America; 5 University of the Chinese Academy of Sciences, Beijing, China; University of Pretoria, South Africa

## Abstract

The usage of invasive tagging methods to assess lizard populations has often been criticised, due to the potential negative effects of marking, which possibly cause increased mortality or altered behaviour. The development of safe, less invasive techniques is essential for improved ecological study and conservation of lizard populations. In this study, we describe a photographic capture-recapture (CR) technique for estimating *Draco dussumieri* (Agamidae) populations. We used photographs of the ventral surface of the patagium to identify individuals. To establish that the naturally occurring blotches remained constant through time, we compared capture and recapture photographs of 45 pen-marked individuals after a 30 day interval. No changes in blotches were observed and individual lizards could be identified with 100% accuracy. The population density of *D. dussumieri* in a two hectare areca-nut plantation was estimated using the CR technique with ten sampling occasions over a ten day period. The resulting recapture histories for 24 individuals were analysed using population models in the program CAPTURE. All models indicated that nearly all individuals were captured. The estimated probability for capturing *D. dussumieri* on at least one occasion was 0.92 and the estimated population density was 13±1.65 lizards/ha. Our results demonstrate the potential for applying CR to population studies in gliding lizards (*Draco spp.*) and other species with distinctive markings.

## Introduction

The ability to recognize individual animals is vital for many wildlife monitoring programmes and behavioural studies [1]. However, marking techniques that alter an animal’s survival probability or other life-history traits violate assumptions of demographic models [2], [3]. Lizards, especially those in the diverse tropical regions have been the subject of relatively few long-term studies, largely because of the difficulties of marking individuals without damaging them. Toe-clipping is a widely used marking technique that requires the removal of a unique combination of digits from each individual [4], [5]. However, toe clipping may affect mobility, particularly for arboreal species. For instance, the clinging ability of the iguanid *Anolis carolinensis* decreased significantly after toe-clipping [6]. Similarly, inflammation was reported one month after toe-clipping in 50% of natterjack toads (*Bufo calamita*) [7]. Yet there are other studies that have reported no effect of toe-clipping in reptiles and amphibians [8]. It is therefore clear that the effects of toe-clipping vary among species and one must carefully test and assess the technique before using it on a large number of individuals [9], [10]. Furthermore, techniques like passive integrated transponders (PIT) [11], tattooing [12] or the bead tagging [13] are not feasible in lizard species with low body mass. The development of a non-invasive technique suitable for assessing populations of small lizards is therefore required.

The photographic capture-recapture (photo-CR) is an easy and inexpensive technique for long-term identification of individuals [14]. Among reptiles it has been used for species with large or medium-sized scales, the shape and size of which are unique to each individual [14]. Scales of most medium to small sized lizards are too small to photograph easily resulting in poor quality photographs, which substantially increase the probability of misclassifying individuals [14], [15]. In a few species of lizards, and some other animals, the ventral surface of the throat and abdomen have blotch-like markings that might be useful for photo-CR, provided these markings can be easily photographed and used to identify individuals reliably.

There are 39 recognised species of gliding lizards (*Draco* spp.) and several new species awaiting description [16]. Because of the aforementioned difficulties in individual recognition the ecology of only a few species has been studied in depth. Although toe-clipping has been used to study *Draco* lizards [17], [18], the treatment effects remain unknown but are suspected to reduce survival and alter behaviour. However, several *Draco* spp. have blotches on the ventral surface of the patagium [16]. Hence, we postulated that photo-CR might offer an opportunity to improve the study of these lizards.

**Figure 1:**
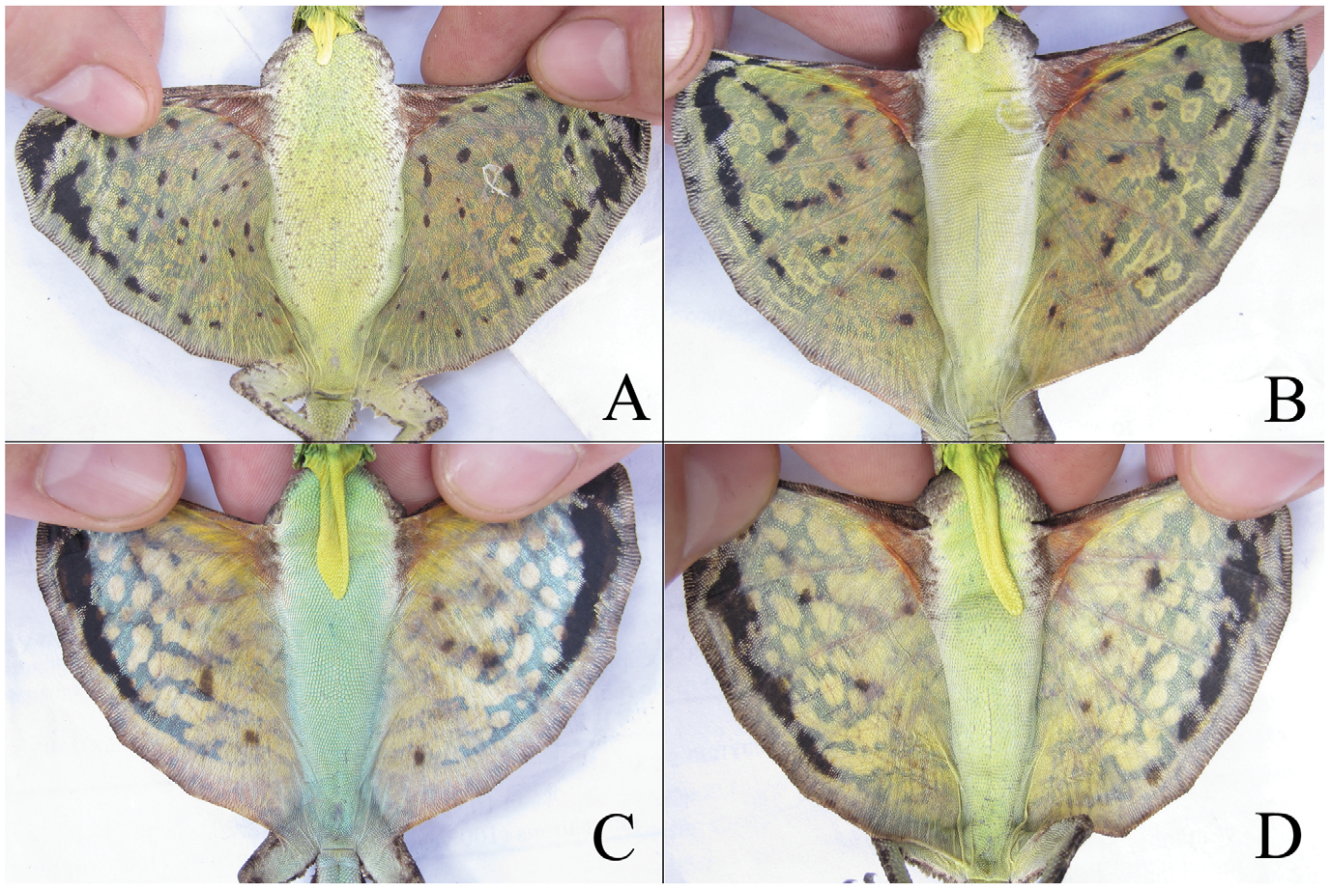
Attributes used to differentiate *Draco dussumieri* individuals. Top: females (with short dewlap), below: males (with long dewlap). The number of blotches on the ventral surface of the patagium in females was significantly greater than that of males.

In this study, we first assess the potential and reliability of photo-CR in studying *D. dussumieri* and subsequently test its practical applicability in estimating population density. *Draco dussumieri* has natural markings on the ventral surface of its gliding membrane (patagium; Figure 1) [19] that are large enough to be photographed and which might be specific to individuals.

## Materials and Methods

### Study Area and Species

The study was conducted in two different privately owned areca-nut (*Areca catechu*) plantations (0.7 and 2 ha respectively) in Agumbe, central Western Ghats, India (13°50′ N, 75°09′ E; 560 m above sea level) with permission from the plantation owners. Both plantations were used for testing the photo-CR technique, with one plantation surveyed per season over two successive seasons. Density was only estimated for the plantation surveyed in the second season.

Agumbe experiences low temperature variation (26–33°C), high humidity (75%–96%) and high rainfall (7,600 mm) [20], [21], most of which is received during the monsoon season (June-September). *Draco dussumieri* is a medium-sized agamid (max. snout-vent length = 97 mm) that is endemic to the hills of southern India. This charismatic animal, though common throughout its range [22]–[24], has been reported only a few times after a series of publications by John between 1962 and 1971 [17], [25]. Since our study area did not fall within a Protected Area, and *Draco dussumieri* is not mentioned in the Wildlife (Protection) Act (1972) and subsequent amendments [26], we did not require government permits to conduct our study. Our study did not entail collection of individuals and we made all efforts to minimize discomfort to the animal by ensuring careful handling and quick release of each captured individual back onto the tree they were first observed on.

### Field Sampling

A team of three conducted the study between April-June in 2010 (first season) and between March-May in 2011 (second season). Lizards were caught by hand after inducing them to glide down from their arboreal perches and to land at the bases of trees or on the ground using long poles [17]. In order to examine whether the blotches on the ventral surface of the patagium remained constant through time, we photographed the patagium and pen-marked 59 individuals dorsally with unique numerical codes using a xylene-free dark-green marker pen. To ensure that all photographs of the same individual were comparable, care was taken while positioning the lizard’s patagium under the camera with minimum body flexing (Figure 1). The area of interest for pattern-matching was below the third rib when counting from the top. Sexes were readily differentiated using the size of dewlap [19], with males possessing distinctively longer dewlaps (Figure 1C, 1D) than females (Figure 1A, 1B). The lizards were then released. After a 30 day interval we recaptured the marked individuals and re-photographed them. Later, we compared the photographs to determine if changes in blotches had occurred between the successive captures.

The density of *D. dussumieri* in the 2 ha areca-nut plantation was estimated in April 2011. The plantation is surrounded by secondary forest on one side and open habitat on the other three sides. The open habitat is unsuitable for *D. dussumieri* and the side of the plantation abutting the secondary forest is separated from the forest by a 17 m wide stream. Thus, the site met the assumptions of closure. Twelve parallel 15 m wide transects were delineated within the plantation and three people simultaneously walked each transect ensuring every tree in the plot was thoroughly inspected. All 12 transects were sampled on each sampling occasion and each sampling occasion was defined by a time-constrained (120 min) walk between 0800 and 1100 hrs. Sampling was conducted over a total of ten occasions over a ten day period. When animals were captured, the time taken to capture and process individuals was subtracted from the survey duration. A short survey period of 10 days ensured negligible probability of any mortality or recruitment occurring during the study period.

### Individual Identification

**Figure 2:**
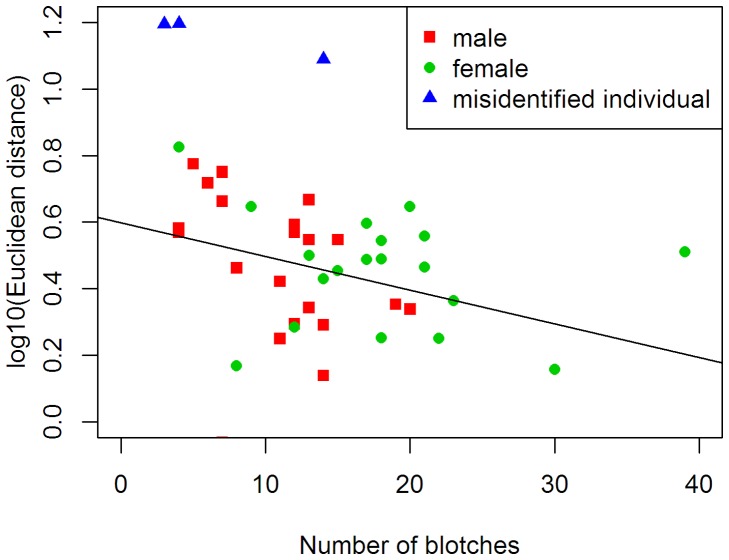
Variation of Euclidean distances in relation to the number of blotches on the ventral side of the patagium. Misidentified individuals were excluded from analysis.

We used the Interactive Individual Identification System (I^3^S Manta) [27] to determine if photo-CR can be used for *D. dussumieri*. A fingerprint file was built by drawing an ellipse around each blotch on the patagium [28]. The software uses a linear transformation to compare two images, so each image is projected onto two-dimensions, irrespective of the individual’s position while photographing it. When comparing two images, a blotch pair is accepted as a good match if the nearest alternative point of a blotch is at least twice the distance of the current match and the ratio between length and width of both the ellipses are sufficiently similar. I^3^S Manta computes a distance metric (Euclidean distance) of images as the sum of the distances between matched points divided by the square of the number of pairs. Further, differences in shape (width to length ratio) and size will also affect the distance metric. A low score in the distance indicates a better match. The distances were log_10_ transformed before analysis to meet the assumptions of normality and homeoscedasticity. We evaluated the effects of the number of blotches to distances using a linear regression after removing the misidentified individuals (outliers) from the data set (Figure 2). Statistical analysis was conducted in the programming and statistical language R 2.13.2 [29].

### Population Density

Capture histories were developed for each individual *D. dussumieri* and analysed using CAPTURE, a program developed to provide population estimates for closed population capture-recapture models [30], [31]. The program computes goodness of fit statistics and a model selection criterion for selecting the best model for the data set in hand [30]. Models implemented by CAPTURE include, individual heterogeneity in capture probabilities (h), behavioural response (b), time (t) and combinations thereof. The null model (M_o_) assumes no variation in capture probabilities among individuals or occasions. We report the estimates computed by CAPTURE for capture probability, abundance and standard-error for abundance for appropriate models. We then used *N/A* as a measure of population density, where *N* is the estimated abundance and *A* is the sampled area. The area of the areca-nut plantation was estimated with geographic-information-methods (ArcGIS 9.3).

## Results

In total, 59 *D. dussumieri* individuals were caught (males = 31, females = 28). Twenty five individuals were captured in the first season and 34 individuals were captured in the second season.

### Photographic Identification Method

We were able to re-capture 45 (76.3%; males = 25; females = 20) marked individuals after a 30-day interval. The Euclidean distance among images decreased with increase in number of blotches on ventral surface of the patagium (*R^2^* = 0.12, df = 39, *P = *0.024; Figure 2). Therefore, a larger number of blotches help the Interactive Individual Identification System (I^3^S Manta) identify individuals more reliably. Females had more blotches in comparison to males (*t* = 4.02, df = 42, *P*<0.005), and I^3^S Manta identified recaptured females with 100% accuracy, whereas males were identified with 88% accuracy. Three individual males were misidentified by I^3^S Manta when used alone, but we could not detect any visual changes in pigmentation pattern on their patagium by eye. A combination of the software and manual identification increased the accuracy of individual identification to 100%.

### Population Density Estimation

There were 74 captures of 24 individuals over the ten sampling occasions (Table 1). The model selection procedure identified the null model (M_o_) as the most appropriate model for the study. Heterogeneity model (M_h_) had the second highest model selection criterion (M_o = _1.00, M_h = _0.91). As we expected *a priori* capture probabilities to be heterogeneous across individuals, we report population size estimates computed under both of these models. Moreover, the population size estimator of model M_h_ is known to be robust to violation of underlying model assumptions [30], [32], [33].

**Table 1:** Capture rates of different *Draco dussumieri* individuals in a 2 ha areca-nut plantation at Agumbe, Western Ghats, India.

Lizard	Capture period										sex	Fi
code	1	2	3	4	5	6	7	8	9	10		
**CM1**	1	1	1	0	0	1	0	0	1	0	M	5
**CFE**	1	1	0	0	0	0	0	0	0	0	F	2
**CM0**	1	0	0	0	0	0	1	0	0	1	M	3
**CF1**	1	0	0	0	0	0	0	0	0	1	F	2
**CM11**	1	0	0	0	0	0	0	0	0	0	M	1
**CM8**	1	1	0	0	1	0	1	0	0	0	M	4
**CFB**	1	0	0	0	0	0	0	0	0	0	F	1
**CM2**	1	1	0	1	0	0	1	1	1	1	M	7
**CM10**	0	1	1	1	0	1	0	0	0	0	M	4
**CM6**	0	1	0	1	1	0	0	0	0	1	M	4
**CM12**	0	1	0	0	0	1	0	1	1	1	M	5
**CM9**	0	1	1	0	0	0	0	0	0	0	M	2
**CM7**	0	0	1	1	1	0	1	1	0	0	M	5
**CM5**	0	0	1	0	0	0	1	0	1	0	M	3
**CF7**	0	0	1	1	1	1	1	1	1	1	F	8
**CFF**	0	0	1	0	0	1	0	1	1	0	F	4
**CF4**	0	0	0	1	1	0	1	0	0	0	F	3
**CM4**	0	0	0	0	1	1	0	0	0	0	M	2
**CF5**	0	0	0	0	1	0	0	0	0	0	F	1
**CF6**	0	0	0	0	0	0	1	0	0	1	F	2
**CM3**	0	0	0	0	0	0	1	1	0	0	M	2
**CFC**	0	0	0	0	0	0	0	1	0	1	F	2
**CMX**	0	0	0	0	0	0	0	0	1	0	M	1
**CMK**	0	0	0	0	0	0	0	0	0	1	M	1

Lizard - *Draco dussumieri,* F_i_ - capture frequency for each animal, M - male, F - female.

Estimated capture probabilities per occasion was 0.30 under model M_o_ and 0.28 under model M_h_. The estimated probability that an individual in the plot was captured on at least one occasion was 1.00 and 0.92 for model M_o_ and M_h_, respectively. In the study site, the abundance estimates under models M_o_ and M_h_ were 24±0.84 (95% confidence interval: 24–29) and 26±3.1 (25–42), respectively. Therefore, the estimated density in a two hectare areca-nut plantation in Agumbe was 12±0.42 (12–14.5) lizards ha^−1^ and 13±1.65 (12.5–21) lizards ha^−1^ for M_o_ and M_h_ model respectively.

## Discussion

A population density of 13±1.65 *D. dussumieri* individuals per hectare in an areca-nut plantation is much higher than the previous estimates of 1.25±0.40 in vanilla plantations [34]. *Draco dussumieri* prefers forested areas [35], one would therefore expect higher densities in plantations that are proximate to natural habitats. Interestingly, this does not explain the drastic difference in the aforementioned density estimates as both studies have been conducted in plantations adjacent to forests. A more probable explanation lies in the major difference in the altitude of both areas: the vanilla plantation was at an altitude between 900–1000 m and the current study was conducted at an elevation of 560 m. A previous study on the encounter rates of agamid lizards in southern Western Ghats showed a sharp decline in the encounter rates of *D. dussumieri* with increasing altitude [24]. Some caution is required in comparing our results with those of Venugopal [34] as the studies used different methods to estimate densities, distance sampling and capture-recapture, respectively.

Photo-CR is a relatively non-invasive and widely accepted method for conducting capture-recapture studies over a broad range of species, and potential alternative to toe-clipping in reptiles [14], [15]. Here, we demonstrate for the first time that the individuals of *D. dussumieri* can be discriminated based on blotches on the ventral surface of their patagium. Moreover, most *Draco* species have similar black blotches on the ventral surface of the patagium [16], suggesting the potential use of photo-CR for identifying individuals in other *Draco* species.

We found that the blotches on the ventral surface of the patagium of *D. dussumieri* did not change (over a 30 day period) and that I^3^S Manta was effective at providing individual identification in combination with manual identification. We assume that the three cases of misclassification by the software resulted largely from the low number of points (blotches) and other user-based errors. For instance, deviation in pointing out the center of the blotches and reference points, variation in the length and width of the ellipse would cause differences in viewing angle and scaling. Users should be aware of these potential sources of error while pointing out the distinguishing features on the photograph. Strict standardization of the image collection process will reduce misclassification.

In *D. dussumieri*, there was a large amount of variation in the number of blotches (range: 3–39) across individuals and I^3^S Manta does not take the number of points (blotches) into consideration while calculating distances. For instance, if two patagium images have ten and thirty blotches respectively, the software may classify them as the same individual if the first ten blotches match without considering the remaining twenty. Another limitation of the software is its ability to illustrate the exact shape of a pattern. The blotches of *D. dussumieri* are irregular and the ellipse shape used by I^3^S Manta does not always accurately illustrate the shape of the blotch. Therefore, in order to avoid misidentification due to these limitations, we suggest the user complement the I^3^S Manta software with a rapid analysis of the most likely matches, which can then be confirmed manually.

The major challenge involved in using photo-CR is the large amount of time needed to digitize individual photographs, which is likely to increase significantly with increasing sample size. Recaptured individuals cannot be readily identified in the field and therefore identification requires photographs and compared with other images in I^3^S Manta. Though pen marks can be used for short-term studies; in rainforests, they often become unreadable after two weeks or are lost during ecdysis. We captured seven individuals with pen-markings up to three months after marking, indicating that the interval between ecdysis can be as long as three months for an adult *D. dussumieri*. The blotches on the ventral surface of the patagium remained the same and we expect that they will remain constant after ecdysis, as blotches are strong and permanent meristic colour patterns. The applicability of photo-CR for long term studies can be further confirmed by using toe-clipping or genetic tagging techniques on a small subset of the study population.

Therefore, photo-CR sampling can be used to obtain a range of behavioural, demographic and ecological factors, which are otherwise difficult to acquire due to lack of proper marking techniques suitable for *Draco* species, and potentially other reptiles with obvious markings.
